# Models and frameworks of patient engagement in health services research: a scoping review protocol

**DOI:** 10.1186/s40900-018-0111-5

**Published:** 2018-09-10

**Authors:** Anna Maria Chudyk, Celeste Waldman, Tara Horrill, Lisa Demczuk, Carolyn Shimmin, Roger Stoddard, Serena Hickes, Annette S. H. Schultz

**Affiliations:** 1 Department of Family Medicine, Rady Faculty of Health Sciences, University of Manitoba, 454-6 - 753 McDermot Avenue, Winnipeg, MB R3E0T6 Canada; 2 College of Nursing, Rady Faculty of Health Sciences, University of Manitoba, 89 Curry Place, Winnipeg, MB R3T 2N2 Canada; 3 Elizabeth Dafoe Library, University of Manitoba, 25 Chancellor’s Circle, Winnipeg, MB R3T 2N2 Canada; 4 George and Fay Yee Centre for Healthcare Innovation, 3rd floor – 753 McDermot Avenue, Winnipeg, MB R3E 0T6 Canada; 5 Horizon Health Network, 80 Woodbridge Street, Fredericton, NB E3B 4R3 Canada; 6 Translating Emergency Knowledge for Kids (TREKK) Parent Advisory Group, Children’s Hospital Research Institute of Manitoba, 512E - 715 McDermot Avenue, Winnipeg, MB R3E 3P4 Canada; 7 College of Nursing, Rady Faculty of Health Sciences, University of Manitoba, CR3022 - 369 Tache Avenue, Winnipeg, MB R2H 2A6 Canada

**Keywords:** Patient engagement, Patient and public involvement, Models, Frameworks, Health services, Scoping review

## Abstract

**Plain English summary:**

Patient engagement in research is an emerging approach that involves active and meaningful collaboration between researchers and patients throughout all phases of a project, including planning, data collection and analysis, and sharing of findings. To better understand the core features (elements) that underlie patient engagement, it is useful to have a look at models and frameworks that guide its conduct. Therefore, this manuscript aims to present a protocol for a scoping review of models and frameworks of patient engagement in health services research. Methods: Our protocol design is based on an established framework for conducting scoping reviews. We will identify relevant models and frameworks through systematic searches of electronic databases, websites, reference lists of included articles, and correspondence with colleagues and experts. We will include published and unpublished articles that present models and frameworks of patient engagement in health services research and exclude those not in English or unavailable as full texts. Two reviewers will independently review abstracts and full texts of identified articles for inclusion and extract relevant data; a third reviewer will resolve discrepancies. Our primary objective is to count and describe elements of patient engagement that overlap (present in 2 or more) and diverge among included models and frameworks. Discussion: We hope this review will raise awareness of existing models and frameworks of patient engagement in health services research. Further, by identifying elements that overlap and diverge between models and frameworks, this review will contribute to a clearer understanding of what patient engagement in research *is* and/or *could be*.

**Abstract:**

Background: Patients can bring an expert voice to healthcare research through their lived experience of receiving healthcare services. Patient engagement in research is an emerging approach that challenges researchers to acknowledge and utilize this expertise through meaningful and active collaboration with patients throughout the research process. In order to facilitate a clearer understanding of the core elements that underlie patient engagement, it is useful to examine existing models and frameworks that guide its conduct. Therefore, the aim of this manuscript is to present a protocol for a scoping review of models and frameworks of patient engagement in health services research. Methods: Drawing on Arksey and O’Malley’s and Levac et al.’s framework for scoping reviews, we designed our protocol to identify relevant a) published articles through systematic searches of 7 electronic databases and snowball sampling and b) unpublished articles through systematic searches of databases and websites and snowball sampling. We will include published and unpublished models and frameworks of patient engagement in health services research and exclude those not in English or unavailable as full texts. Two reviewers will independently screen the abstracts and full texts of identified articles for inclusion and extract relevant data; a third reviewer will resolve disagreements. We will conduct a descriptive analysis of the characteristics (i.e., elements underlying patient engagement and those related to the study authors, publication, and model/framework) of included articles and a narrative analysis of the data concerning elements of the model or framework. Our primary objective is to count and describe elements of patient engagement that overlap (present in ≥ 2) and diverge (present in < 2) among identified models and frameworks. Discussion: Through identification of elements that overlap and diverge between existing models and frameworks, this review will provide a starting point for the critical reflection on our collective understanding of what patient engagement in health services research *is* and/or *could be*. Ultimately, we hope that the findings of this review raise awareness of existing models and frameworks and shed light on some of the complexity of conducting patient engaged research through identification of key elements that shape this approach.

## Background

Patients with lived experience of receiving healthcare services can bring an expert voice to healthcare research. Their expertise lies in their lived experience, which may offer unique insight into healthcare service experiences, how these services affect their health, how services support them to take control of their own health, and systemic and geographical barriers to healthcare access [1]. Patient engagement is a research approach that involves meaningful and active collaboration between researchers and patients in governance, priority setting, research conduct and knowledge translation [2]. It is often described as researchers doing research with patients, rather than for or on them, through integration of patients into the role of study team member [3] (herein referred to as partnership). There is growing interest in using patient engagement to integrate the expert voice of patients into health services research. There is also a growing demand for patient engagement in research from funders.

Initiatives that encourage patient engagement in research have emerged fairly recently across North America and Europe. Within Canada, for example, the Canadian Institutes of Health Research’s Strategy for Patient Oriented Research (SPOR) aims to build capacity for a continuum of research that engages patients as partners, focuses on patient identified priorities, and improves patient outcomes [4]. Since 2011, the Canadian federal government has allocated over $60.5 million (CAN) to SPOR; this has helped support activities such as grant funding calls and the establishment of Support for People and Patient-Oriented Research and Trials (SUPPORT) Units across provinces and territories [5]. Alternatively, in the United States (US) in 2010, the Patient-Centered Outcomes Research Institute (PCORI) was established as part of the US’s Patient Protection and Affordable Care Act [6]. PCORI is committed to the funding, production and promotion of clinical research guided by patients, caregivers, and the healthcare community. Finally, in 1996, the United Kingdom’s National Institutes for Health Research (NIHR) established a national advisory group (INVOLVE) to support active public involvement in National Health Service, public health and social care research [7]. INVOLVE activities include publication of resources for researchers, establishment of a library of resources, and leading NIHR projects focused on public involvement in research. Initiatives such as SPOR, PCORI, and INVOLVE help provide the financial, structural, and educational support necessary for patient engagement in research.

In the field, researchers play a critical role in partnering with patients and families to support them in their new roles as research partners. Research leads, in particular, are integral to the development, conduct and dissemination of research; they also facilitate relationship management among the research team. With the inclusion of patients as research partners, as opposed to research participants/subjects, the role of team management expands. The challenge can be working with team members who come with experiential knowledge of relevance to the study but possibly limited insight into research processes. These different knowledge bases are located within power structures that surround research endeavors – within academic institutions, levels of education, social locations, as examples [8]. For a two-way exchange of information to occur, all team members need to feel empowered to contribute knowledge.

Models and frameworks contribute to the development of knowledge through systematic description of a phenomenon of interest [9]. Within the field of patient engagement, models and frameworks provide a richer understanding of the core elements that underlie patient engagement, which potentially include the relationships among and flow of information between researchers, patients, and other stakeholders. They are a powerful tool for researchers to reflect upon and move away from the standard approach to research in which patients are participants more so than partners. To help researchers and patients better understand their newfound research partnerships, it is useful to examine existing models and frameworks that guide their conduct. To our knowledge, there are no scoping reviews of models and frameworks of patient engagement in health services research. Such a review is necessary to develop a clearer understanding of the core elements that underlie patient engagement in research and inform guidelines on how to conduct this research. This will also serve to address prevailing criticisms of patient engagement in research, which include lack of an agreed upon model or framework to guide its conduct and, more generally, lack of awareness among researchers of frameworks that guide this work [10, 11].

### Aims

The aim of this manuscript is to present a protocol for a scoping review of models and frameworks of patient engagement in health services research.

### Team composition

Our scoping review draws on the experiences and expertise of eight individuals. The lead author (AC) is a post-doctoral fellow whose current research focuses on the development of scholarship surrounding patient engagement in health services research. The second author (CW) was a nursing doctoral student and her graduate course work laid the foundation for this study; she herself was involved in research as a patient partner. The third author (TH) is a doctoral student and comes with a nursing background. Our team also includes an expert health librarian (LD), a patient engagement research expert (CS), and two individuals (RS and SH) with experience in the role of patient partner in research studies. The final author (AS) is a senior scientist and advisor of the post-doctoral fellow and doctoral students. She comes with considerable experience in conducting scoping reviews and her program of research includes active involvement with a variety of team members. Each of these authors come with expert knowledge of relevance to our scoping review and their roles will create space for expression of their knowledge and experiences.

## Methods/design

A scoping review is a type of knowledge synthesis that addresses an exploratory research question aimed at mapping key concepts (elements), types of evidence, and gaps in research [12]. It involves systematic searches for, and selection and synthesis of, existing knowledge across a range of study designs [12, 13]. Thus, unlike a systematic review, there is typically no requirement to weigh the evidence according to a formal assessment of methodological quality [13]. We propose to undertake a scoping review that aims to a) identify and describe models and frameworks of patient engagement in health services research and b) describe elements that overlap and diverge among models and frameworks of patient engagement in health services research. Ahead of conducting this scoping review, we publish this protocol in accordance with the Preferred Reporting Items for Systematic Review and Meta-Analysis Protocols (PRISMA-P) 2015 Statement [14]. A review protocol documents the planned methodological approach to a review’s conduct (prior to the review being carried out) [15]. Review protocols are important because they allow reviewers to carefully plan and thereby anticipate potential problems and avoid arbitrary decision making when conducting a review; enable others to identify selective reporting, replicate methods, and judge the validity of proposed methods; and reduce the duplication of reviews on the same topic while potentially prompting collaborations [15]. The design of this protocol is based on the seminal work by Arksey and O’Malley [16] and enhancements to this work by Levac et al. [12, 17]. Accordingly, we organized this protocol into six stages, which we expand upon below.

### Stage 1: Identification of the research question

The first stage involves identification of a clearly articulated a) broad research question that acts as a roadmap for subsequent stages and b) scope of inquiry (i.e., definition of the concept, target population, and outcomes of interest) that assists in the identification and selection of studies in subsequent stages. We undertake this coming review to answer the following research question:

*What are the elements that underlie models and frameworks of patient engagement in health services research?*

Key concepts within our research question include “models”, “frameworks”, “patient engagement”, and “health services research.” We define these concepts in Table 1. Models and frameworks are related but distinct concepts, so we expand upon the definitions we provide in Table 1, here. A framework provides the conceptual underpinnings of a study by providing a general list of variables (elements) that comprise, and therefore should be used to analyze, a phenomenon of interest [9]. It aims to describe and identify the universal elements that comprise a phenomenon of interest without actually specifying the relations among these elements [9]. In contrast, a model is developed within a framework, and attempts to describe and simplify a phenomenon of interest through the loose description of relations between elements [9].

**Table 1:** Definitions of key concepts within our research question

Concept	Definition
Models	A descriptive and deliberate simplification of a phenomenon of interest or an aspect of a phenomenon of interest [9].
Frameworks	A shared orientation for studying, explaining, and understanding phenomena of interest through the description and identification of the universal elements underlying a phenomenon of interest [9].
Patient engagement	Meaningful and active collaboration of patients in research governance, priority setting, conduct, and knowledge translation [2].
Health services research	The study of how social factors, financing systems, organizational structures and processes, health technologies, and personal behaviours affect access to health care, the quality and cost of health care, and, ultimately, the population’s health and well-being. It includes research with the goal of improving the efficiency and effectiveness of health professionals and the health care system, through changes to practice and policy [21].

Our target population includes all studies that present a model and/or framework of patient engagement in health services research. Our envisioned outcome is a description of the elements that overlap and diverge between identified models and frameworks, as well as a descriptive summary of the studies that we identify; this descriptive summary will provide context to our findings of elements that overlap and diverge between models and frameworks.

### Stage 2: Identification of relevant studies

This stage involves balancing the breadth and depth of the scoping review with feasibility.

#### Search methods

We propose to search the following electronic databases: CINAHL, Cochrane Database of Systematic Reviews, Joanna Briggs Institute Evidence Based Practice Database, Medline, PsycInfo, and Scopus, as well as Google Scholar. We will use a comprehensive search strategy that utilizes search terms (key words and/or medical subject headings or subject headings) that relate to our key concepts, combines search terms within a concept with the Boolean term ‘OR’, combines search terms between concepts with the Boolean term ‘AND’, and is adapted to the syntax used by each database. An expert librarian (LD) will collaborate with the first author (AC) to develop the search strategy. We present a sample search strategy in Table 2. Based upon consultation with LD, we plan to screen only the first 10 pages of results obtained from Google Scholar for inclusion. We will additionally search for unpublished models and frameworks through electronic database (i.e., ProQuest Dissertations & Theses, Conference Proceedings Citation Index) and website (i.e., PCORI, Google) searches. We will follow up our search of electronic databases and websites with snowball sampling [18]; this includes backward and forward reference searching of included articles and correspondence with colleagues and experts in the field about relevant models and frameworks.

**Table 2:** Sample search strategy for Medline (Ovid)

Search number	Search terms
1	Patient Participation/
2	(patient adj3 engag*).mp
3	(“patient and public involvement” or “patient involvement” or “stakeholder engagement”).mp
4	models, theoretical/ or patient-specific modeling/
5	(model or models or framework*).mp
6	research.mp
7	(“patient oriented research”).mp
8	1 or 2 or 3
9	4 or 5
10	6 or 7
11	8 and 9 and 10

#### Inclusion/exclusion criteria

We will include published and unpublished models and frameworks of patient engagement in health services research. We will rely on the labels attached by the original study authors to identify and differentiate between models and frameworks. For clarity, we specify that we will exclude models and frameworks that do not focus on health services research; for example, those related to patient engagement in clinical decision-making/practice, active role of patients in health management, understanding disease experience, therapeutic or technology engagement, pharmaceutical research etc. We will also exclude articles that are unavailable as full texts, and due to feasibility related- reasons (e.g., limited resources, including funding to hire translators), those that are not published in English.

### Stage 3: Study selection

We will use a four-step process for study selection, using the inclusion/exclusion criteria identified in Stage 2:

1. A single reviewer (AC) will screen the eligibility of article titles identified through database searches, Google Scholar, and searches for unpublished models and frameworks. The reviewer will record whether an article is included or excluded and the primary reason for exclusion (where applicable).
2. Two reviewers (AC and TH) will independently screen the eligibility of abstracts of articles included in Step 1. Each reviewer will note whether an article is included or excluded and the primary reason for exclusion (where applicable). Reviewers will meet upon completion to compare results and resolve discrepancies. If they cannot resolve a discrepancy, they will consult with a third reviewer (AS) to reach consensus. Study selection is an iterative process. As such, when they meet, reviewers will also discuss challenges and uncertainties that arise during study selection and refine the search strategy if needed based on the input of the entire research team.
3. Two independent reviewers will screen the eligibility of full texts of articles included in Step 2, using the protocol described in Step 2.
4. We will follow up our search of electronic databases and websites with snowball sampling, using the process outlined in Steps 1–3. This will determine the final set of included articles.

**Fig. 1:**
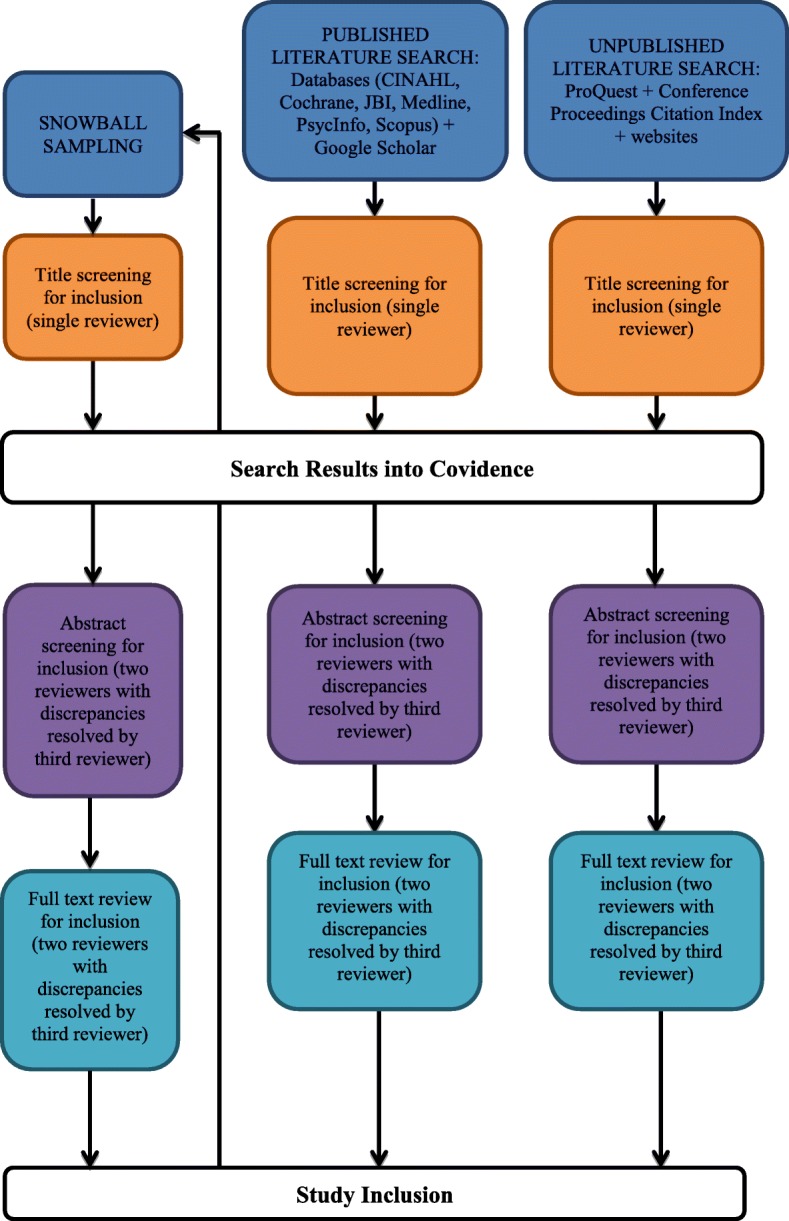
Flow of studies within Stages 2 and 3

We propose to use Covidence (Veritas Health Innovation, Melbourne Australia) to manage study selection between reviewers. Covidence is an online application designed to aid with the production and management of systematic reviews [19]. It is the standard production platform for Cochrane Reviews. Figure 1 displays the flow of studies within Stages 2 and 3.

### Stage 4: Charting (extracting) the data

Two reviewers (AC and TH) will independently extract data from the full texts of articles included in the review. They will use a standardized extraction form jointly developed by the research team. We display a list of variables that we propose to extract, by over-arching category, in Table 3.

**Table 3:** Variables that we propose to extract, by over-arching category

Category	Variable
Characteristics of the study authors	Surnames
	Countries (that authors’ primary institutional affiliations are located in)
	Lens/disciplinary points of view
Characteristics of the publication	Study name
	Publication year
	Journal
	Published (yes/no)
Characteristics of the model/framework	Name
	Purpose/aim
	Population developed in/for
	Targeted stage of research process
	Elements (description)^a^
	Proposed relations between elements^a^
	Location on spectrum of engagement [22]
	Strengths and weaknesses (stated in study)

^a^These data directly address our research question

For each included article, we initially propose to extract the a) descriptions of elements that underlie patient engagement, as well as b) any proposed relations between these elements. These data directly correspond to our stated research question. In addition, we propose to extract general descriptive data specific to the study authors, publication and model/framework to provide context and to support a richer interpretation of our findings of elements that overlap and diverge between models and frameworks of patient engagement in health services research. Reviewers (AC and TH) will meet with the senior author (AS) twice during this process -- after they extract data from 5 to 10 studies and again after they have extracted data from all of the studies. At these meetings, they will discuss any need to modify the data extraction form and document rationale for any proposed modifications. They will only make changes to the data extraction form after consultation with the entire research team.

### Stage 5: Collating, summarizing and reporting the results

This stage involves considerations related to data analysis, reporting of results, and implications of findings. We will provide a descriptive analysis of the characteristics (i.e., elements that underlie patient engagement, study authors, publication, and model/framework) of included articles, as well as a narrative analysis of the data. Our primary objective is to count and describe elements of patient engagement that overlap and diverge between identified models and frameworks. We define overlap as elements that are present in 2 or more models or frameworks and diverge as elements that are present in less than 2 models or frameworks of patient engagement in health services research. Initial presentation of extracted data will be to the research team, and elements will be labeled using language found in the original articles. Through consultation with the research team, we may collapse elements into over-arching elements. We envision that these findings will have implications for research and practice, as well as patients and researchers that choose to engage in research as partners. For example, this review will make readers more aware of existing models and frameworks that they can use to guide their work. It will also identify commonalities in how existing models and frameworks conceptualize patient engagement in health services research. This serves as a solid starting point for the understanding of what patient engagement in health services research *is*. That said, we acknowledge that patient engagement in researcher does not represent a “one size fits all” approach to research. We will therefore also synthesize diverging elements that a given reader may decide are appropriate for his/her study, project, or general understanding of patient engagement in research.

### Stage 6: Consultation

This stage is optional in Arksey and O’Malley’s original framework for conducting scoping reviews [16], whereas Levac et al. [17] recommend that this stage is an essential component of scoping review methodology. It focuses on development of a plan to consult with stakeholders about study findings and the dissemination of these findings. We propose to consult with two stakeholders (RS and SH) that have served as patient partners on 1 or more research projects. The purpose and goals of consultation are to integrate the experiences of patient partners to ensure that the design, conduct, and knowledge translation of a scoping review is relevant to the population it involves – researchers and patient partners. As such, our stakeholders are valued members of our research team and the term ‘engagement’ (used herein instead of collaboration) more accurately describes their involvement [2]. Our primary method of engagement will be small-group meetings that involve sharing of information and discussions to maximize the likelihood of an environment that supports contribution from all team members [20]. At the outset of partnership, the research co-leads (AC and AS) and patient partners (RS and SH) developed a terms of reference for patient engagement to support active and meaningful collaboration on the project. The terms of reference provides a brief overview of the project, identifies the names and positions of research team members and the researcher appointed to the role of patient partner - researcher liaison, and the nature of the relationships and expectations between patient partners and researchers. It is a “living document” that the authors will re-visit and revise as necessary throughout the duration of their partnership. We expand upon the subsections of this document in Table 4.

**Table 4:** Terms of reference: Subsections and key components within each

Subsection	Key components
Preamble	Brief background and project aim(s).
The research team	Team members’ names and positions.
Responsibilities and opportunities for patient partners	General expectations (e.g., communicate feedback, concerns, requests for accommodations, etc. to the patient partner-researcher liaison; review documents ahead of and participate in meetings; provide timely feedback, etc.) and stages of the research process that patient partners will be involved in and associated major tasks.
Responsibilities and opportunities for patient partner – researcher liaison	Frequency and nature of contact with patient partners and mechanisms that help ensure patient partners feel included, heard and valued.
Responsibilities and opportunities for researchers	General expectations related to patient engagement (e.g., recognize lived experience as a form of knowledge and expertise, be mindful of wording for any written materials, maintain a fair and structured relationship that does not cross professional boundaries, etc.).
Process (work plan)	General project related responsibilities of the other team members, patient partners’ preferred modes of feedback, frequency of full-team meetings.
Expected outcomes	Major project milestones (e.g., proposed manuscripts, presentations, potential future projects, etc.).
Compensation (for patient partners)	Amount that patient partners will be compensated for project-related activities (e.g., meeting participation, transportation, and time spent preparing for meetings, reviewing and providing feedback on study documents, etc.) and preferred types of compensation (e.g., monetary, gift cards to specific vendors, reimbursement for registration to courses/workshops, etc.).

## Discussion

Patient engagement challenges researchers to reflect upon and potentially change how they approach research. Specifically, researchers may need to reconsider previous learnings about the construction of expertise and the value of experiential knowledge, as well as their approach to the flow of information and decision-making between patients and researchers. Its criticisms include lack of an agreed upon model or framework to guide its conduct and, more generally, lack of awareness among researchers of frameworks that guide this work [10, 11]. To help foster its growth and development, now is a good time to reflect on how the published and unpublished literature conceptualizes this research approach. To our knowledge, this is the first scoping review of models and frameworks of patient engagement in health services research. Findings of this scoping review will provide the opportunity to reflect critically upon patient engagement in research and support both researchers and patients in a richer understanding of their new roles as research partners. Through identification of elements that overlap and diverge between existing models and frameworks, this review provides a starting point for the critical reflection on our collective understanding of what patient engagement in health services research *is* and/or *could be*. Since patient partners are a heterogeneous group that represents diverse perspectives and experiences, and research projects that engage with patients often contain innovative and unique aspects, a unifying model or framework of patient engagement may not be a realistic or appropriate goal. Therefore, ultimately, we hope that the findings of this review raise awareness of existing models and frameworks and shed light on some of the complexity of conducting patient-engaged research through identification of key elements that shape this approach.

## Abbreviations

NIHR: National Institutes for Health Research
PCORI: Patient-Centered Outcomes Research Institute
PRISMA-P: Preferred Reporting Items for Systematic Review and Meta-Analysis Protocols
SPOR: Strategy for Patient Oriented Research
SUPPORT: Support for People and Patient-Oriented Research and Trials
US: United States
